# Effect of Salt Solution Environment on the Aging of Styrene−Butadiene−Styrene (SBS)-Modified Asphalt

**DOI:** 10.3390/polym16121709

**Published:** 2024-06-14

**Authors:** Chengwei Xing, Bohan Zhu, Kingsley C. K. Chiang, Cheng Chen, Lingxiao Liu, Zhibin Chang

**Affiliations:** 1 Key Laboratory for Special Area Highway Engineering of Ministry of Education, Chang’an University, South 2nd Ring Road Middle Section, Xi’an 710064, China; xingcw@chd.edu.cn (C.X.);; 2 School of Highway, Chang’an University, South 2nd Ring Road Middle Section, Xi’an 710064, China; 3 China State Construction Engineering (Hong Kong) Limited, Hong Kong 999077, China

**Keywords:** SBS-modified asphalt, aging, salt solution environment, rheological properties

## Abstract

The aim of this paper is to investigate the aging mechanism of asphalt in the sea salt erosion environment from a rheological point of view. In order to simulate the real pavement aging process in the sea salt erosion environment, base asphalt and Styrene−Butadiene−Styrene (SBS)-modified asphalt were selected for salt environment aging tests. The asphalt samples were aged via a thin film oven test (TFOT) and a pressure aging vessel (PAV) test. Then, thermo-oxidizing conditions were created after the samples were immersed in salt solution, mixed with four different concentrations of sodium chloride (NaCl) and sodium sulphate (Na_2_SO_4_), to investigate the aging state of asphalt. Temperature scan (TS), frequency scan (FS), and multiple stress creep and recovery (MSCR) tests performed using a Dynamic Shear Rheometer (DSR) were used to investigate the effects on the rheological properties of aged asphalt in a salt environment. The results showed that both base asphalt and SBS-modified asphalt were aged to different degrees under mixed salt solutions. The two asphalt samples aged in a salt environment showed increased hardness. SBS-modified asphalt exhibited higher aging resistance compared with base asphalt in the sea salt environment. However, due to the degradation of the SBS modifier and the aging of base asphalt, the properties of the SBS-modified asphalt showed more obvious complexity with changes in salt solution concentrations.

## 1. Introduction

Nowadays, improving the durability of pavements and promoting the development of green highways are the focuses of road engineering research [1,2]. Asphalt, as the main material for road paving, is aged under the impact of its environment and traffic loads, which reduces certain properties of the asphalt and shortens the service lives of pavements [3,4,5]. SBS, as an excellent asphalt modifier, can enhance the high-temperature rutting resistance and low-temperature cracking resistance of asphalt, and, therefore, it is widely used in the construction of high-grade pavements [6,7,8]. Several scholars studied the aging behavior of SBS-modified asphalt in thermo-oxidative, ultraviolet, and freeze–thaw environments [4,9,10,11]. The aging process mainly consists of the base asphalt aging and the SBS modifier degrading [9]. In addition, asphalt pavement in some areas is often affected by salty environments, such as the industrial salt used to dissolve snow on roads in cold areas and seawater immersion in coastal areas. Therefore, it is important to investigate the effects of salty environments on asphalt pavement aging [12,13,14].

In view of this, some scholars made a comparative analysis of the physical and chemical properties of asphalt before and after being aged in salt solution. Ogbon et al. [15] immersed virgin base asphalt (PNE 30/60) in different concentrations of NaCl solution and proved, through the three major index tests and rheological tests, that the aging of the asphalt deepened with the increase in salt solution concentration. Wei et al. [16] placed 70# virgin base asphalt in NaCl solution for aging tests, and found that the causes of asphalt aging included not only the thermal and oxygen environment but also the salt environment. After being aged, the high-temperature resistance of asphalt was enhanced, the light components of the micro-components changed to heavy components, and the contents of aromatic hydrocarbons and gels were reduced. Similarly, by using 70# virgin base asphalt in NaCl solution, Zhang et al. [17] found that the salt environment aggravated the aging degree of asphalt.

Several scholars also investigated the aging behavior of SBS-modified asphalt in NaCl solution. For example, Zhang et al. [13] and Ogbon et al. [15] reported that the SBS modifier could reduce the aging effects of salt solution on asphalt. Wei et al. [16] further found that the aging change patterns of SBS-modified asphalt were similar to those of base asphalt, but the aging process of SBS-modified asphalt in the salt environment included the degradation of SBS in addition to base asphalt aging. An investigation by Guo et al. [18] into the property changes in SBS-modified asphalt in a salt environment showed that salt solution effects on asphalt properties could be attributed to salt aging and the physical actions of salt particles. Additionally, road property tests showed that the water stability, high-temperature properties, and low-temperature properties of asphalt mixtures first decreased and then leveled off as the salt solution concentration increased.

All of the above scholars used a salt solution made from a single NaCl for the purpose of simulating a salt environment, but studies have revealed that the salt composition of seawater contains more than 80% chlorine salts and more than 10% sulfate salts [19,20]. Consequently, Zhang et al. [19] used the mixed salt solution of NaCl and Na_2_SO_4_ in a mass ratio of 8:1 for immersion tests. The results showed that salt particles would move into the interior of the asphalt, thus reducing its stability and decreasing the surface free energy and cohesive work of aged asphalt. The viscosity of SBS-modified asphalt decreased and tended to evolve in the elastic direction, thus enhancing the high-temperature rutting resistance and decreasing the low-temperature cracking resistance. The aging degree was positively correlated with the salt solution concentration. Subsequently, Zhang et al. [20] further investigated the aging of SBS-modified asphalt and its corresponding mixtures in a mixed solution of NaCl and Na_2_SO_4_. By investigating the roles of dry and wet cycles, freezing cycles, and thawing cycles in these experiments, it was concluded that the high-temperature sensitivity of asphalt was strengthened after salt environment erosion, while the low-temperature cracking resistance was significantly damaged. Moreover, sea salt could enter the asphalt binder and cause the deterioration of the asphalt. Meanwhile, it was found that salt particles in the mixed salt solution were transferred to the asphalt mixtures and accumulated on the asphalt surface. The split tensile strength and water stability of asphalt mixtures were severely damaged, which was related to the salt concentration and the number of freeze–thaw cycles.

The above studies made up for the lack of authenticity in the sea salt environment used by earlier scholars. However, a large number of salt crystals adhering to the soaked asphalt surface will also affect the rheological properties of asphalt, thus affecting the judgment of the salt environment effects on asphalt aging behavior. In addition, the actual pavement is affected by sea salt erosion, as well as heat and oxygen, so it is necessary to consider the effects of heat, oxygen, and the salt environment on SBS-modified asphalt. Therefore, to address the above problems, this study compares the aging differences between base asphalt and SBS-modified asphalt in a combined thermal–oxygen and sea salt environment, using rheological tests in order to clarify the aging mechanism of SBS-modified asphalt in the salt environment. Specifically, TS, FS, and MSCR tests were used to evaluate the rheological indices of base asphalt and SBS-modified asphalt. The rutting factor, aging factor, average recovery rate index, and so on were calculated by analyzing the obtained results, which were used to analyze the specific rheological condition of aged asphalt in a salt environment.

## 2. Materials and Methods

### 2.1. Materials

In this study, virgin base asphalt with a penetration of 80–100 was used, to which a SBS modifier accounting for approximately 4% of the asphalt mass was applied. Adding SBS improved the base properties of the asphalt, allowing SBS-modified asphalt to meet the requirements of the JTG F40-2004 [21] in China. The technical conditions of the NaCl and Na_2_SO_4_ used in this study were in accordance with GB/T 1266-2006 [22] and GB/T 9853-2008 [23], respectively.

### 2.2. Preparation of Salt Solution

The mixed salt manufactured during the tests was a mixture of NaCl crystals and anhydrous Na_2_SO_4_ crystals in the ratio of 8:1 by mass. The mixed salt particles were weighed at 0% (pure water), 6%, 12%, and 18% of the clear water mass, respectively, to configure the mixed salt solution of the corresponding concentration.

### 2.3. Aging Specimens Preparation

#### 2.3.1. TFOT

In accordance with the American Society for Testing and Materials (ASTM) standard D1754 [24], virgin base asphalt and virgin SBS-modified asphalt were placed in a thin-film oven to simulate the short-term aging of asphalt that occurred during preparation, transportation, and paving. During the experiments, 50 g ± 0.5 g of virgin base asphalt or virgin SBS-modified asphalt was uniformly spread in an aging pan with a diameter of 140 mm ± 0.5 mm and a thickness of about 3.2 mm. Then, it was placed in a thin-film oven for 5 h at 15 ± 0.2 r/min and 163 °C.

#### 2.3.2. PAV Test

After being short-term aged, base asphalt and SBS-modified asphalt were subjected to the PAV test in accordance with the ASTM Standard D6521 [25]. The PAV can simulate the long-term aging of the pavement caused by heavy loads, wind, and rain in the course of service. The aging pans containing asphalt were placed in PAV at 100 °C and 2.1 MPa for 20 h. The aging samples were mixed at the end of the tests to minimize variability errors.

#### 2.3.3. Salt Environment Aging Test

**Figure 1:**
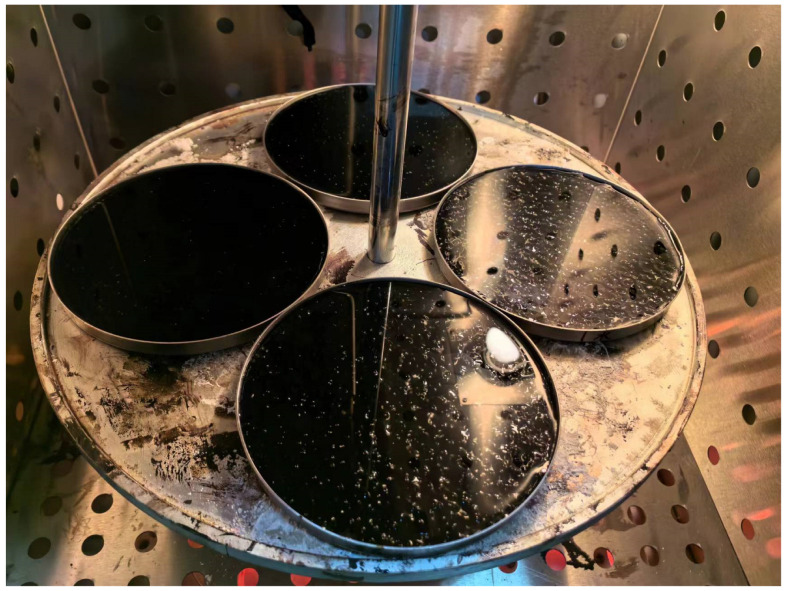
The aging process of asphalt in a salt environment.

After short-term and long-term aging, the asphalt was placed in the aging pans covered with the mixture of 60 g NaCl and Na_2_SO_4_ for aging in a sea salt environment. Then, the asphalt samples were placed in the thin-film oven at 95 °C, 15 ± 0.2 r/min, for 12 h, as shown in Figure 1. After salt aging, the aging pans were removed and placed in water for 12 h to dissolve the salt particles on the surface of the asphalt. This was used as a cycle to perform four cycles for each sample in the experiment. For illustration purposes, the abbreviations defining the different asphalt are shown in Table 1.

### 2.4. Test Methods

#### 2.4.1. Salt Solution Volatilization Rate Test

The volatilization rates of salt solution at different concentrations were determined using a constant temperature oven to compare the differences in volatilization rates caused by different salt concentrations. The oven temperature was set to 150 °C to minimize the test time. Each salt solution of 50 g with concentrations of 0%, 6%, 12%, and 18% was simultaneously placed in a constant temperature oven at 150 °C. Their residual mass was tested every 10 min for a total of 50 min.

#### 2.4.2. TS Test

The TS test is a multi-temperature rheological property test conducted with the DSR. According to the ASTM standard D7175 [26], the test rotor used a parallel plate with a diameter of 25 mm and a gap of 1 mm. The test temperature was controlled as 46–76 °C, with a step of 6 °C as the condition to determine the complex modulus (G*) and phase angle (δ) of asphalt at different temperatures. Then, the complex modulus aging index (CMAI) and phase angle aging index (PAAI) were defined to evaluate the aging degree.

(1) $$CMAI=\frac{G_{age}^{*}}{G_{V}^{*}}$$

(2) $$PAAI=\frac{\delta_{age}}{\delta_{V}}$$

#### 2.4.3. FS Test

FS is a rheological property test based on the DSR. The temperature range used in the test was 40–70 °C with a temperature interval of 10 °C. A rotor with a diameter of 25 mm and a gap of 1 mm was used when the temperature was higher than 30 °C. After the linear viscoelastic test, the shear strain and the frequency were set to 1% and 0.1 rad/s–100 rad/s, respectively. The results of the frequency scanning test were used to plot the Cole–Cole plots and Black plots.

#### 2.4.4. MSCR Test

The MSCR test is also a DSR-based test used to evaluate the rutting resistance of asphalt at high temperatures. These tests were conducted at 58 °C and 64 °C, respectively, in accordance with AASHTO T350 [27]. The samples were loaded for 1 s and recovered for 9 s as a cycle for a total of 30 cycles. The first 20 cycles were loaded with 0.1 KPa, while the last 10 cycles were loaded with 3.2 KPa. Data were collected from the 10th to the 30th cycle of the tests, which were used to calculate the key evaluation parameters: recovery rate (R) and non-recoverable creep compliance (Jnr).

## 3. Results and Discussion

### 3.1. Salt Solution Volatilization Rate

**Figure 2:**
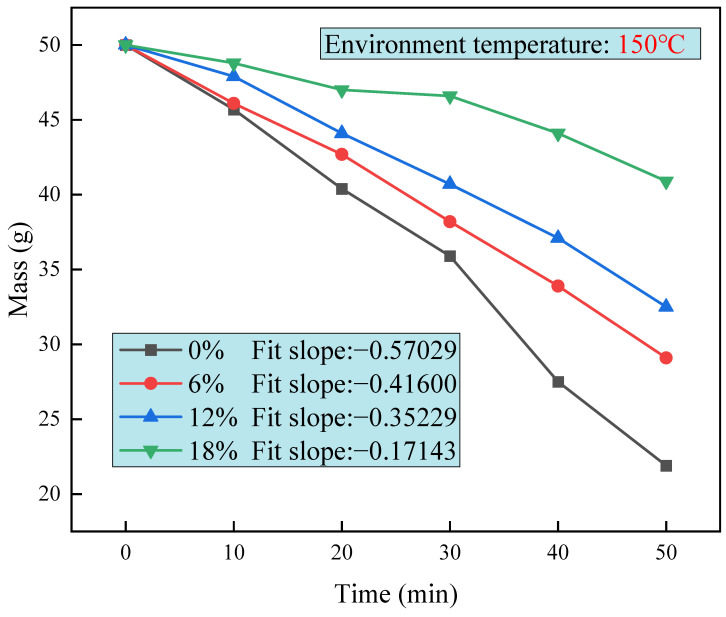
Volatilization rates of salt solutions at different concentrations.

The comparison of the salt solution volatilization at different concentrations can be visualized in Figure 2, which shows that the volatilization rates between clear water and salt solution are different. From the overall qualitative analysis, it can be deduced that the mass reduction per ten minutes of solution decreases significantly with the increase in the salt solution concentration. From the quantitative analysis, a linear fit was made to each folded line in Figure 2, with the slope of the fit representing the volatilization rate of the solution per unit of time. The salt solution with a concentration of 18% shows a 70% reduction in the volatilization rate compared with water. This is because the water molecules in the brine have to overcome the binding force with the salt mixture when volatilizing. The higher the salt solution concentration, the greater the binding force.

### 3.2. Analysis for TS Test

#### 3.2.1. Rutting Factor

**Figure 3:**
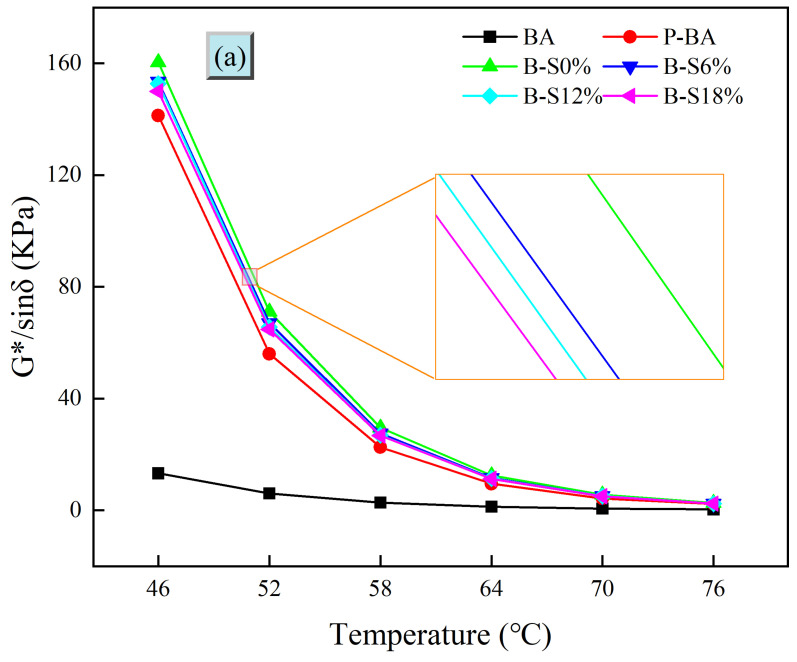

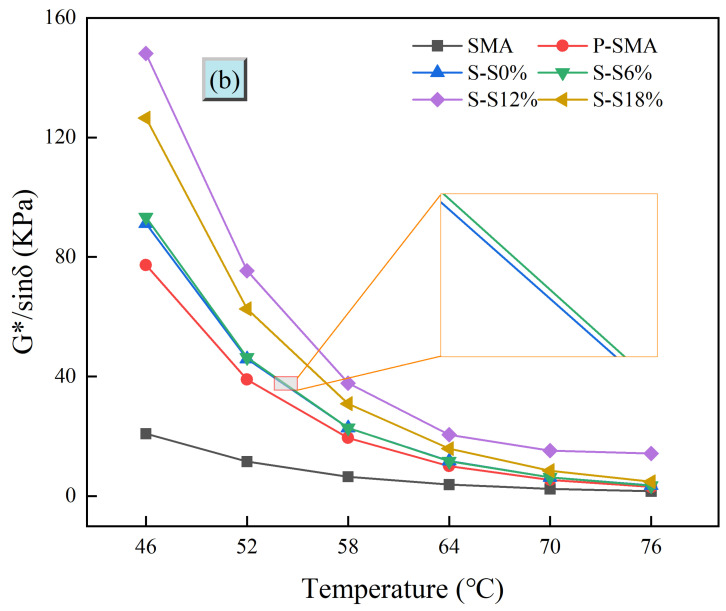
Trends of rutting factors of different asphalt at different temperatures: (**a**) base asphalt; (**b**) SBS-modified asphalt.

The rutting factor (G*/sinδ) was calculated by the complex modulus and phase angle from the TS test, reflecting the high-temperature rutting resistance of asphalt. The rutting factor changes in base asphalt and SBS-modified asphalt after being aged are shown in Figure 3. First, from the aging process of base asphalt (Figure 3a), it can be clearly seen that the rutting factor value of the virgin base asphalt changes in a smaller magnitude and gradually tends to stabilize with the increase in temperature. From 46 °C to 76 °C, the rutting factor of virgin base asphalt decreases by only 2%. After short-term and long-term aging, the rutting factor of the asphalt increases dramatically by as much as 900% when the temperature is 46 °C. Under the coupled action of thermo-oxidative and high pressure, the light components in virgin base asphalt volatilized, and the aromatic components transformed to colloidal and then to asphaltene, thus leading to the rapid hardening of virgin base asphalt [28]. The rutting factor of P-BA also decreases rapidly with increasing temperature and eventually levels off. There is a significant increase in the rutting factor of the asphalt with the addition of the mixed salt solution. Interestingly, the hardening of asphalt is the highest with the salt concentration of 0%. As the salt concentration increases from 6% to 18%, the rutting factor of asphalt decreases by 2%. This is because the asphalt is subjected to the coupled effects of salt environmental and thermo-oxidative aging. As mentioned above, the volatilization rate of salt solution is much smaller than that of water. Although salt accelerated the aging of asphalt in the previous research [13], the samples in the aging tests were completely covered by salt water or clear water, which act as the oxygen barrier. As the salt solution concentration increased, it evaporated more slowly, so the asphalt was subjected to less thermo-oxidative aging. The degree of salt environmental aging was lower than that of thermo-oxidative aging, which resulted in lower asphalt aging degree.

In Figure 3b, it can be seen that the rutting factor of asphalt increases dramatically after thermo-oxidative and pressure aging, but the rutting factor of P-SMA is 46% smaller than that of P-BA at 46 °C, which indicates that SBS-modified asphalt has better resistance to aging compared with base asphalt. The rutting factors of SBS-modified asphalt are enhanced by 17% and 20% compared with P-SMA at 46 °C when salt solution is added at concentrations of 0% and 6%, respectively. It can be seen that salt solution aggravates the aging of SBS-modified asphalt, but the low concentration of salt solution has little effect. The rutting factor of SBS-modified asphalt increases by 91% compared with P-SMA as the salt solution concentration increases to 12%. It indicates that at this concentration, the salt erosion effects on SBS-modified asphalt increase significantly. When the salt solution concentration increases to 18%, the rutting factor of SBS-modified asphalt is smaller than that of S-S12%. The reason for this phenomenon may be that the concentration of 18% salt solution is more difficult to volatilize, so the SBS-modified asphalt has the shortest contact time with oxygen, resulting in a lower degree of SBS degradation and base asphalt aging.

In summary, SBS-modified asphalt is less affected by salt solution than base asphalt. However, SBS-modified asphalt is more sensitive to salt solution concentration effects. The main reason may be that SBS degradation in salt environment and the coupled effects of thermal and oxygen influence lead to the more complex aging of SBS-modified asphalt.

#### 3.2.2. Aging Index

**Figure 4:**
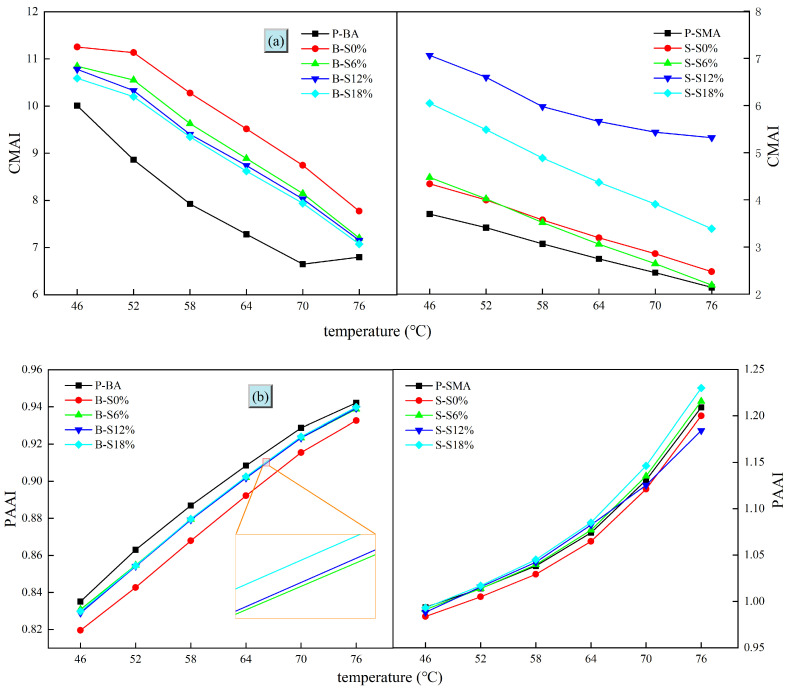
Variations in the aging indexes of different asphalts at different temperatures: (**a**) CMAI; (**b**) PAAI.

The aging indexes of base asphalt and SBS-modified asphalt in different environments are shown in Figure 4. It should be noted that the higher the CMAI, the lower the PAAI, indicating that the aging index is higher. As seen in Figure 4a, the change rule of CMAI is similar to that of the rutting factor. The aging degree of SBS-modified asphalt under different aging environments is lower than that of base asphalt, showing that the crosslinked structure of SBS can improve the densification degree of asphalt and effectively reduce the aging effects of thermo-oxidative or salt. In Figure 4a, it can be seen that SBS-modified asphalt is more easily affected by salt solution, and the aging degree is the highest when the salt solution concentration is 12%. As for PAAI shown in Figure 4b, the smaller its value is, the higher the aging degree of asphalt. The change rule of PAAI of base asphalt is similar to that of CMAI, but the change rule of SBS-modified asphalt is more complicated. According to Figure 4a,b, the CMAI values of base asphalt are all greater than those of SBS-modified asphalt. The values of PAAI are all less than those pf SBS-modified asphalt, which indicates that the aging index of base asphalt is greater. Overall, the aging indexes can clearly express the change law of base asphalt and SBS-modified asphalt aging in different conditions. The SBS-modified asphalt aging index is significantly lower than that of base asphalt, which means that SBS modification can reduce the corrosion of salt solution on base asphalt. However, it can also be seen that SBS-modified asphalt is more sensitive to the influence of salt solution than base asphalt.

### 3.3. Analysis for FS Test

#### 3.3.1. Cole–Cole Diagram

**Figure 5:**
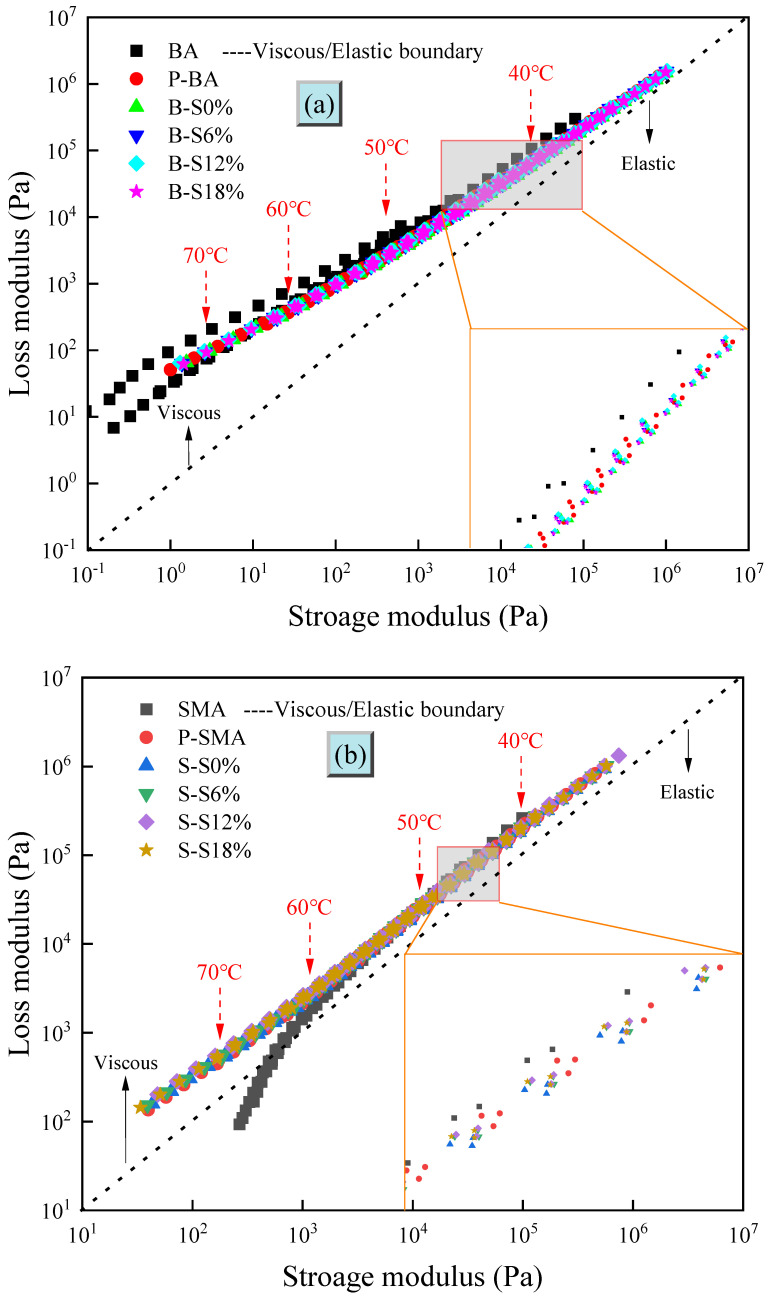
Cole–Cole diagram of various asphalts: (**a**) base asphalt; (**b**) SBS-modified asphalt.

Figure 5 shows the Cole–Cole diagrams of base asphalt and SBS-modified asphalt aged under different conditions, where the diagonal line divides the properties of asphalt into elastic-dominated and viscous-dominated intervals [29]. When the modulus curve is above the diagonal line, the asphalt behaves as viscous and vice versa as elastic [30]. The relationship between elastic and viscous of asphalt at different temperatures is illustrated in Figure 5. Firstly, it can be seen in Figure 5a that the loss modulus and storage modulus of virgin base asphalt gradually decrease with increasing temperature under different aging conditions. All the samples are basically above the diagonal line, showing their viscous nature. The scatter plots of P-BA are closer to the diagonal line compared with virgin base asphalt because the light components in the aged asphalt volatilize and the asphalt hardens. The asphalt with a salt solution concentration of 0% is the closest to the diagonal line, indicating that the aged asphalt has the lowest viscosity. As the concentration of salt solution increases, the viscosity increases. The modulus of SBS-modified asphalt with different aging degrees similarly decreases with increasing temperature. All the samples are in the viscous range except for only a portion of the virgin SBS-modified asphalt that is in the elastic range. The modulus changes in SBS-modified asphalt in different aging forms are more complex, it can be seen the base asphalt gradually moves away from the diagonal direction as the temperature increases, but SBS-modified asphalt changes insignificantly, indicating that base asphalt has higher temperature sensitivity [31].

#### 3.3.2. Black Space Diagram

**Figure 6:**
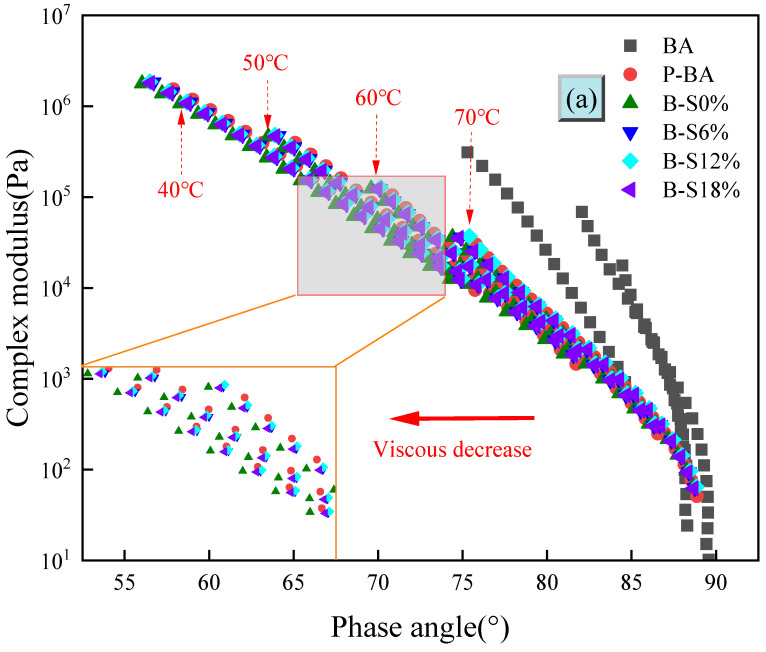

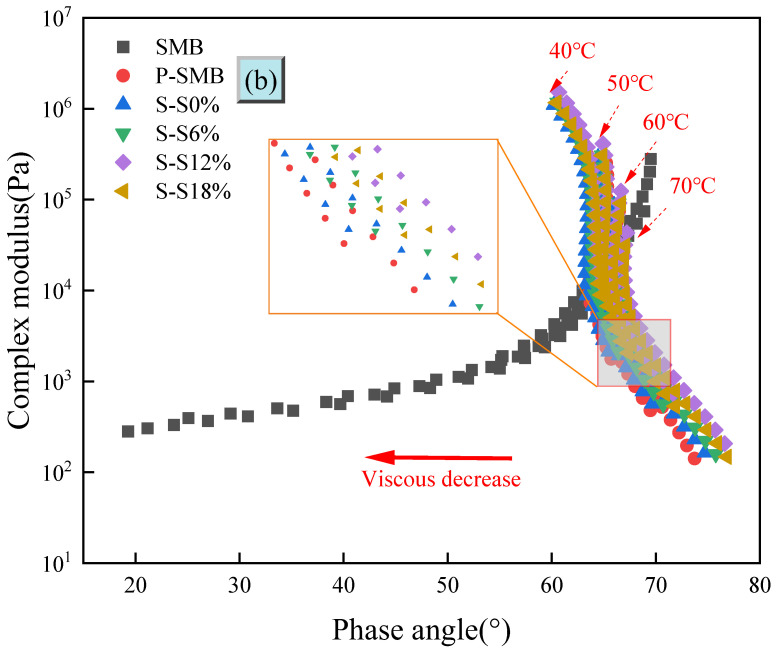
Black space diagram of different asphalt: (**a**) base asphalt; (**b**) SBS-modified asphalt.

Based on the complex shear modulus and phase angle acquired from the FS test, the black space diagram was plotted to analyze the changing law of aged asphalt viscoelasticity [32]. As shown in Figure 6a, the phase angle of virgin base asphalt is close to 90° when the complex modulus is small [33]. The aged asphalt shifts to the left overall, with the left curve corresponding to a more hardened asphalt. As the temperature decreases, the elastic properties become more prominent. It can be seen in Figure 6a that virgin base asphalt aged by a salt solution concentration of 0% exhibits more pronounced elastic properties, which correspond to the temperature scan results.

It can be seen from Figure 6b that the black space diagram of the SBS-modified asphalt is quite different from that of base asphalt. SBS-modified asphalt has a larger range of variation in the phase angle due to the stabilized crosslinked structure provided by the SBS modifier, which allows the viscoelastic range of the modified asphalt to be varied to a greater extent. The black space diagram of SBS-modified asphalt changes a lot before and after aging, which is not like base asphalt, showing obvious changes in the patterns of test results at different temperatures. This is due to the spatial crosslinking structure of the SBS modifier. In the high-frequency zone, the phase angle of the virgin SBS-modified asphalt is larger. The asphalt scatter plot moves to the left after being aged in salt solution. In the low-frequency region, the phase angle of virgin SBS-modified asphalt is smaller. The asphalt scatter plot moves to the right after being aged in salt solution. Furthermore, the aging degree of SBS-modified asphalt increases with increasing salt concentration, indicating that SBS-modified asphalt is more sensitive to the salt aging effects.

### 3.4. Analysis for MSCR Test

#### 3.4.1. Shear Strain Curve

**Figure 7:**
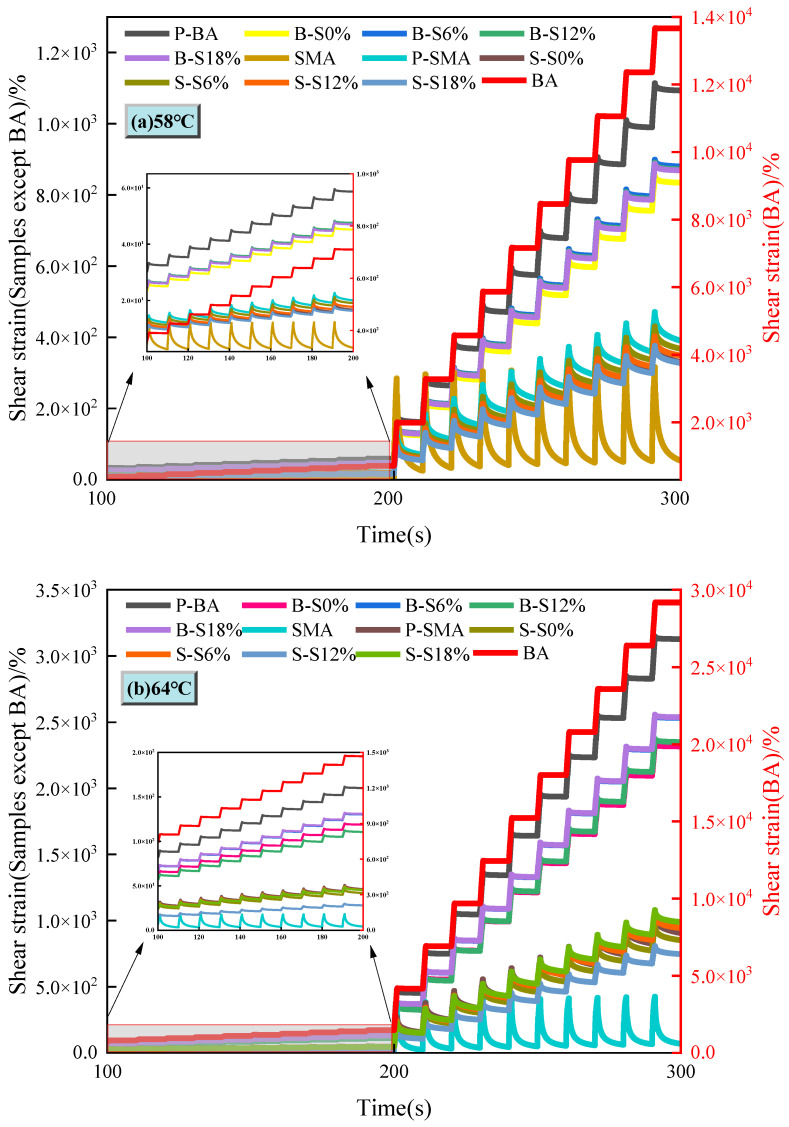
MSCR shear strains of different asphalts at different temperatures: (**a**) 58 °C; (**b**) 64 °C.

**Figure 8:**
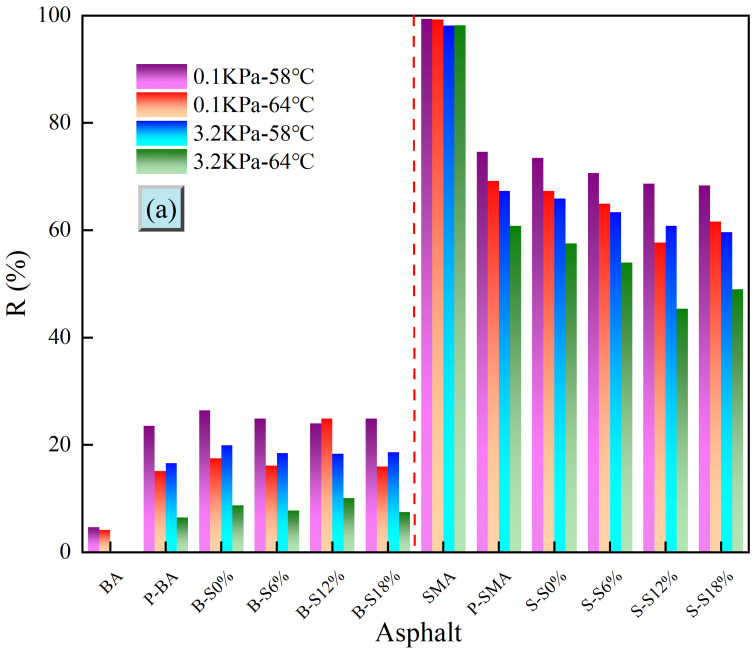

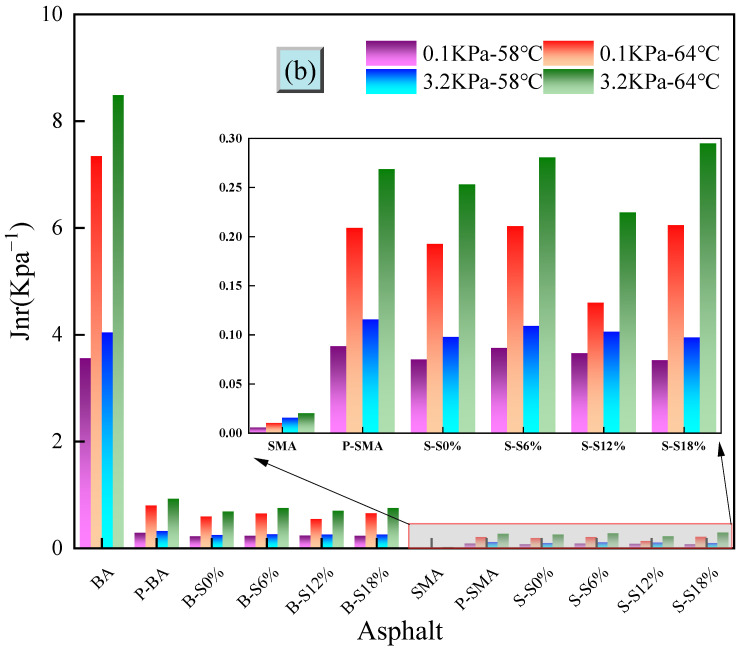
R and Jnr of base asphalt and SBS-modified asphalt: (**a**) R; (**b**) Jnr.

The changes in shear strain of asphalt at different aging conditions at 58 °C and 64 °C are shown in Figure 7. The asphalt samples exhibit different strain change phenomena at 0.1 KPa and 3.2 Kpa, respectively. Regardless of whether the temperature is 58 °C or 64 °C, the strain amplitude of virgin base asphalt is much larger than that of aged asphalt. Therefore, Figure 7a,b use two vertical coordinates to represent the shear strain of virgin base asphalt (right side, BA) and other asphalt samples (left side, samples except BA), respectively. The shear strains of virgin base asphalt reach 1.366 × 10^4^% and 2.92 × 10^4^% at 58 °C and 64 °C (3.2 KPa), respectively. In contrast, virgin SBS-modified asphalt shows the best recovery effect, with strains of only 54% and 69% at 300 s at 58 °C and 64 °C (3.2 KPa). The maximum strains of base asphalt after PAV aging are reduced by 92% and 89% at both temperatures (3.2 KPa). It can be seen that the deformation resistance of aged base asphalt is greatly improved. Similarly, the deformation resistance of base asphalt after being aged by salt solution is further improved. It can be seen that the base asphalt without salt solution has the best resistance to deformation. The deformation resistance of base asphalt is slightly reduced by increasing the solution concentration. In comparison, SBS-modified asphalt still maintains good deformation and recovery ability after aging. It can be clearly seen in Figure 8 that the deformation resistance of aged SBS-modified asphalt is better than that of aged base asphalt. Furthermore, the deformation resistance of SBS-modified asphalt after aging by salt solution is higher than that of the samples after thermo-oxygen and pressure aging, indicating that salt solution has a promotional effect on SBS-modified asphalt aging. Through a comprehensive comparison of base asphalt and SBS-modified asphalt aging laws in different salt solutions, it can be seen that SBS-modified asphalt is more complexly affected by salt solution because of SBS degradation and bas e asphalt aging.

#### 3.4.2. R and Jnr

R and Jnr can be used to quantitatively analyze the deformation resistance of asphalt at different temperatures and under different aging environments, as shown in Figure 8. In Figure 8a,b, it can be seen that the Rs of virgin base asphalt and corresponding aged asphalt are much smaller than that of SBS-modified asphalt. The Rs of the two kinds of asphalt at 0.1 KPa are much larger than those at 3.2 KPa, which is because the large stress causes the greater permanent deformation of the asphalt, while SBS provides the stable crosslinked structure required to resist permanent deformation caused by stress. When the temperature increases from 58 °C to 64 °C, the Rs of different stresses and types of asphalt decrease, indicating that high temperature reduces the permanent deformation resistance of asphalt. The R value of virgin SBS-modified asphalt is greater than 90%, indicating that virgin SBS-modified asphalt has a high recovery ability after receiving loads. The R of aged SBS-modified asphalt is above 40%, while those of base asphalt and aged base asphalt do not exceed 30%, which further indicates that SBS can effectively improve the permanent deformation resistance of asphalt. The R of base asphalt increases after thermo-oxidative and pressure aging, which further increases with the addition of salt solution. However, the R is maximum when the salt solution concentration is 0%, which is similar to the previous conclusion. In contrast, the R of SBS-modified asphalt decreases sharply after thermo-oxidative and pressure aging, which is due to the effects of SBS degradation and base asphalt aging. The degradation of SBS weakens the deformation resistance of asphalt, which leads to a significant decrease in the R of P-SMA. After being aged by salt solution, the R of SBS-modified asphalt is still much larger than that of base asphalt. The change in R is complicated.

There is a similar reflection in Figure 8b. The Jnr of aged SBS-modified asphalt is significantly smaller than that of base asphalt, meaning that the SBS-modified asphalt after salt aging still has better resistance to deformation. Moreover, the Jnr of SBS-modified asphalt is smallest when the salt solution concentration is 12%, which indicates that the asphalt has the best high-temperature rutting resistance. This corresponds to the results of the above test and also shows the complexity of the SBS-modified asphalt aging situation in salt solution.

## 4. Conclusions

In this paper, base asphalt and SBS-modified asphalt were aged in thermo-oxygen, pressure, and salt environments to compare the changes in properties, thus leading to the study of the salt solution effects on asphalt aging. The rheological properties of aged asphalt were investigated using rheological tests in order to draw the following conclusions:

(1) The salt solution has a smaller volatilization rate than the water solution at the same temperature. Moreover, the greater the concentration of the salt solution, the smaller the volatilization rate.
(2) The rutting factors of SBS-modified asphalt and base asphalt after aging in salt solution are significantly enhanced. The rutting factors of base asphalt after aging are higher than that of SBS-modified asphalt, indicating that SBS-modified asphalt has better resistance to aging. After the coupled aging effects of salt solution and oxygen, the aging degree of base asphalt in salt solution is lower than that of pure water, and SBS-modified asphalt shows a higher degree of aging when the salt solution concentration is 12%. Similar conclusions are obtained using aging index analysis.
(3) The Cole–Cole diagrams in the FS test results indicate that with the increase in temperature, the SBS modifiers can effectively prevent the viscosity of asphalt from increasing after salt solution aging. From the black space diagram, it can be seen that the changes in SBS-modified asphalt after aging in salt solution are significantly different in the high-frequency region and low-frequency region. This indicates that the viscoelastic change in SBS-modified asphalt after aging is more complicated than that of base asphalt.
(4) The recovery ability of SBS-modified asphalt after salt solution aging is much larger than that of base asphalt. SBS-modified asphalt has the smallest Jnr and the greatest resistance to rutting at high temperatures at a salt solution concentration of 12. This also shows that the complexity of SBS-modified asphalt changes with the aging condition of salt solution.

## Figures and Tables

**Table 1:** Abbreviations corresponding to asphalt samples.

Number	Terminology	Abbreviate
1	Virgin base asphalt	BA
2	Virgin SBS-modified asphalt	SMA
3	Long-term aged base asphalt	P-BA
4	Long-term aged SBS-modified asphalt	P-SMA
5	0% Salt + Long-term aged base asphalt	B-S0%
6	6% Salt + Long-term aged base asphalt	B-S6%
7	12% Salt + Long-term aged base asphalt	B-S12%
8	18% Salt + Long-term aged base asphalt	B-S18%
9	0% Salt + Long-term aged SBS-modified asphalt	S-S0%
10	6% Salt + Long-term aged SBS-modified asphalt	S-S6%
11	12% Salt + Long-term aged SBS-modified asphalt	S-S12%
12	18% Salt + Long-term aged SBS-modified asphalt	S-S18%

## Data Availability

The data presented in this study are available on request from the corresponding author.

## References

[1] Xing C.W., Li M.C., Liu L.Y., Lu R., Liu N., Wu W.J., Yuan D.D. (2023). A comprehensive review on the blending condition between virgin and RAP asphalt binders in hot recycled asphalt mixtures: Mechanisms, evaluation methods, and influencing factors. J. Clean. Prod..

[2] Office J.E., Cavalli M.C., Chen D., Chen Q., Chen Y., Falchetto A.C., Fang M., Gu H., Han Z., He Z. (2023). Review of advanced road materials, structures, equipment, and detection technologies. J. Road Eng..

[3] Yu H., Ma T., Wang D., Wang Z., Lv S., Zhu X.Y., Liu P., Li F., Xiao Y., Zhang J. (2020). Review on China’s Pavement Engineering Research2020. Zhongguo Gonglu Xuebao/China J. Highw. Transp..

[4] Wang W., Yang L., Cui H., Wu F., Cheng Y., Liang C. (2023). Freeze–Thaw Damage Mechanism Analysis of SBS Asphalt Mixture Containing Basalt Fiber and Lignocellulosic Fiber Based on Microscopic Void Characteristics. Polymers.

[5] Yu H., Bai X., Qian G., Wei H., Gong X., Jin J., Li Z. (2019). Impact of Ultraviolet Radiation on the Aging Properties of SBS-Modified Asphalt Binders. Polymers.

[6] Zaidullin I.M., Petrova L.M., Yakubov M.R., Borisov D.N. (2013). Variation of the composition of asphaltenes in the course of bitumen aging in the presence of antioxidants. Russ. J. Appl. Chem..

[7] Wu W.J., Cavalli M.C., Jiang W., Kringos N. (2024). Differing perspectives on the use of high-content SBS polymer-modified bitumen. Constr. Build. Mater..

[8] Wu W., Jiang W., Xiao J., Yuan D., Wang T., Ling X. (2024). Investigation of LAS-based fatigue evaluation methods for high-viscosity modified asphalt binders with high-content polymers. Constr. Build. Mater..

[9] Xing C.W., Jiang W., Wang M., Zhao K., Li Z.H. (2023). Minireview on the Rejuvenation of Aged Styrene-Butadiene-Styrene (SBS) Modified Bitumen: State-of-the-Art and Outlook. Energy Fuels.

[10] Liu G., Nielsen E., Komacka J., Greet L., van de Ven M. (2014). Rheological and chemical evaluation on the ageing properties of SBS polymer modified bitumen: From the laboratory to the field. Constr. Build. Mater..

[11] Liu L., Dong W., Sun L., Jiang T. (2009). Ultraviolet radiation aging performance of SBS and SBR modified asphalt. J. Build. Mater..

[12] Zhang Q., Wu D.L., Zhang X.J., Chang K.L., Wang Y.B. (2021). Effect of organic deicing agents on asphalt rheology and analysis of the mechanism. Constr. Build. Mater..

[13] Zhang Q.L., Huang Z.Y. (2019). Investigation of the Microcharacteristics of Asphalt Mastics under Dry-Wet and Freeze-Thaw Cycles in a Coastal Salt Environment. Materials.

[14] Feng D., Yi J., Wang D., Chen L. (2010). Impact of salt and freeze–thaw cycles on performance of asphalt mixtures in coastal frozen region of China. Cold Reg. Sci. Technol..

[15] Ogbon W.A., Xu H.N., Jiang W., Xing C.W. (2023). Polymer-modified asphalt binders’ properties deterioration under the action of chloride salt. Road Mater. Pavement Des..

[16] Wei H., Bai X.P., Qian G.P., Wang F.Y., Li Z.F., Jin J., Zhang Y.H. (2019). Aging Mechanism and Properties of SBS Modified Bitumen under Complex Environmental Conditions. Materials.

[17] Zhang Y.H., Wei H., Dai Y.H. (2020). Influence of Different Aging Environments on Rheological Behavior and Structural Properties of Rubber Asphalt. Materials.

[18] Guo R.H., Zhang H.H., Tan Y.X. (2022). Influence of salt dissolution on durable performance of asphalt and Self-ice-melting asphalt mixture. Constr. Build. Mater..

[19] Zhang R., Tang N.P., Zhu H.Z. (2022). The effect of sea salt solution erosion on cohesion, chemical and rheological properties of SBS modified asphalt. Constr. Build. Mater..

[20] Zhang R., Tang N.P., Deng X.K., Zhu H.Z., Su C.L., Xi Y. (2022). Erosion mechanism of sea salt solution on the performance of SBS-modified asphalt mixtures. Int. J. Pavement Eng..

[21] (2004). Technical Specifications for Construction of Highway Asphalt Pavements.

[22] (2006). Chemical Reagents. Sodium Chloride.

[23] (2008). Chemical Reagent. Sodium Sulfate Anhydrous.

[24] (2002). Standard Test Method for Effect of Heat and Air on Asphaltic Materials (Thin-Film Oven Test).

[25] (2022). Standard Practice for Accelerated Aging of Asphalt Binder Using a Pressurized Aging Vessel (PAV).

[26] (2015). Standard Test Method for Determining the Rheological Properties of Asphalt Binder Using a Dynamic Shear Rheometer.

[27] (2019). Standard Method of Test for Multiple Stress Creep Recovery (MSCR) Test of Asphalt Binder Using a Dynamic Shear Rheometer (DSR).

[28] Xing C.W., Liu L.P., Cui Y., Ding D. (2020). Analysis of base bitumen chemical composition and aging behaviors via atomic force microscopy-based infrared spectroscopy. Fuel.

[29] Tarar M.A., Khan A.H., Rehman Z.U., Inam A. (2021). Changes in the rheological characteristics of asphalt binders modified with soybean-derived materials. Int. J. Pavement Eng..

[30] Wu W.J., Jiang W., Xiao J.J., Yuan D.D., Wang T., Xing C.W. (2022). Analysis of thermal susceptibility and rheological properties of asphalt binder modified with microwave activated crumb rubber. J. Clean. Prod..

[31] Shi K., Ma F., Liu J., Song R.M., Fu Z., Dai J.S., Li C., Wen Y.L. (2022). Development of a new rejuvenator for aged SBS modified asphalt binder. J. Clean. Prod..

[32] Tarar M.A., Khan A.H., Rehman Z.U., Qamar S., Akhtar M.N. (2020). Performance characteristics of asphalt binders modified with sunflower flour: A sustainable application of renewable resource derived material. Constr. Build. Mater..

[33] Airey G.D. (2002). Use of black diagrams to identify inconsistencies in rheological data. Road Mater. Pavement Des..

